# Anchovy powder enrichment in brown rice‐based instant cereal: a process optimization study using Response Surface Methodology (RSM)

**DOI:** 10.1002/fsn3.2424

**Published:** 2021-06-30

**Authors:** Paa T. Akonor, Amy Atter, Margaret Owusu, Jonathan Ampah, Anthonia Andoh‐Odoom, Ragnhild Overå, Marian Kjellevold, Johannes Pucher, Jeppe Kolding

**Affiliations:** ^1^ CSIR‐Food Research Institute Accra Ghana; ^2^ Department of Geography University of Bergen Bergen Norway; ^3^ Institute of Marine Research Bergen Norway; ^4^ German Federal Institute for Risk Assessment Berlin Germany; ^5^ Department of Biological Sciences University of Bergen High Technology Center Bergen Norway

**Keywords:** anchovies, brown rice, drum drying, instant cereal, optimization, small fish

## Abstract

There is a need for expanding the utilization of small fish as they constitute an undervalued and important source of protein and micronutrients in many developing countries suffering from micronutrient deficiencies. One way to increase consumption and health benefits is to add nutrient‐rich fish meal into staple food ingredients. In this study, Response Surface Methodology (RSM) was applied to optimize the processing of an instant rice‐based cereal enriched with anchovy powder. The Box‐Behnken design was used to study the effect of principal processing variables (drying temperature, drum rotation speed, and slurry solids concentration) on product water activity, color, bulk density, and water solubility index. Viscosity, consistency, and cohesiveness of the reconstitute cereal were also evaluated. Empirical models were developed to describe the relationship between independent and dependent variables and showed regression coefficients (*R* ^2^) ranging between 71% and 98%. Higher drying temperatures resulted in reduced water activity, darker product color, and lower consistency. While drum speed influenced (*p* < .05) product color and water‐binding capacity, bulk density, and consistency of the reconstituted product was associated with slurry solids concentration. Optimal processing conditions obtained from the study were temperature of 130°C, drum speed of 9.3 rpm, and solids concentration of 20.5%. These conditions would be useful in the production of brown rice‐based instant cereal enriched with anchovy powder with desired quality properties.

## Graphical abstract

One way to increase fish consumption is to add fish meal into staple food ingredients. Response Surface Methodology (RSM) was applied to optimize the processing of an instant cereal enriched with anchovy powder. Optimal processing conditions were at the temperature of 130°C, drum speed of 9.3 rpm, and slurry solids concentration of 20.5%.

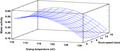

## 1 INTRODUCTION

Breakfast is the most important meal of the day (Gibney et al., 2018). Breakfast meals are mostly based on major cereals and other staples peculiar to different geographical locations. In Africa and many parts of the developing world, rice is among the leading cereals for making breakfast porridges. Although white rice is the most preferred (Selvam et al., 2017), brown rice has a superior nutritional profile and contains important bioactive compounds with potential health benefits, and therefore, its consumption is being recommended (Mir et al., 2017; Rosniyana et al., 2006). Brown rice contains 7.1%–8.3% crude protein, 1.6%–2.8% fat, 1.0%–1.5% ash, and 0.6%–1.0% fiber (Saleh et al., 2019) and provides ample amounts of vitamins, minerals, and amino acids (Liu et al., 2009). Early mornings, preparing for the various chores of the day, are busy times in most families, and quick and easy meals are often preferred. Given this background, brown rice could serve as a potential higher nutritional raw material to produce instant rice‐based foods such as porridges.

Attempts to enhance the nutritional profile of cereal porridges have relied on using legumes to improve the protein quality by providing essential amino acids (Temba et al., 2016). This approach has gained traction in the fight against malnutrition by providing satisfactory nutrients to consumers. In this context, many blends containing either wheat, rice, maize, sorghum, or millet in combination with soybean or cowpea, and peanuts have been studied (Asma et al., 2006; Ejigui et al., 2007; Eshun et al., 2011). Mayachiew et al. (2015) developed an instant extruded rice porridge powder containing either soybean or mung bean. A recent study by Eshun (2019) considered a blend of rice, sesame seeds and cowpea in the development of a drum‐dried complementary food. Notwithstanding the reasonable quality of protein offered by plant‐based blends, protein of animal origin, including fish, has superior amino acid pattern and higher biological value.

Fish is an important source of good quality protein and important micronutrients such as calcium, zinc, iodine, vitamin A and B_12_, and‐very long‐chain polyunsaturated fatty acids (VLCPUFA) such as eicosapentaenoic acid (EPA, 20:5n‐3) and docosahexaenoic acid (DHA, 22:6n‐3) (Aakre et al., 2020; Hasselberg et al., 2020; Reksten et al., 2020). Micronutrient deficiency (hidden hunger) limits growth, mental development, health, and working capacity (Elvevoll & James, 2000). Small pelagic fish such as herrings, sardines, anchovies, and mackerel species constitute one‐third of the global fish production and the harvest is the most environmentally friendly animal‐sourced food production system available (Herrick et al., 2010, Hilborn et al., 2018; Lazar et al., 2018). Anchovies is a nutrient‐dense food commodity (Hasselberg et al., 2020; Sankar et al., 2013), and utilization of anchovies and other fish species in complimentary diets and other instant porridges will enhance the overall quality of protein and micronutrient content. Additionally, there is a need for improved utilization of small fish, which is also used as fish meal in animal nutrition instead of human food (Kumolu‐Johnson & Ndimele, 2011).

Drum drying is a major hydrothermal processing method for processing instant cereals. This drying technique is suitable for viscous suspensions or slurries in food processing (Daud, 2006). In drum drying, the food which is in slurry form is spread over the surface of a rotating heated drum. It is during the rotation action that drying occurs. Generally, the overall thermal efficiencies of drum dryers range from 35% to 80% (Earle and Earle, 2004). It has been used in the production of instant starches, dairy products, geriatric foods, and instant cereals. Drum‐dried foods are convenient, require minimum preparation prior to consumption due to their precooked nature. Additionally, drum‐dried foods easily reconstitute into a pasty mass of soft consistency which is easy to eat, and therefore, often used in breakfast meals.

Response surface methodology (RSM) is a mathematical and statistical technique used to derive model equations that determine a specific response whose outcome is influenced by many variables and describe the relationship between them. This is a useful tool for efficiently exploring and optimizing processes (Madamba, 2002). RSM has been widely used in the optimization of many food processes (Afoakwa et al., 2006; Annor et al., 2010; Madamba, 2002; Sobukola et al., 2010). Given the fact that small pelagic fish, such as anchovies, is a good source of protein and micronutrient (Hasselberg et al., 2020), it could be used in combination with cereals in the production of breakfast meals, including instant porridges. This will contribute to diversifying the utilization potential of anchovies, and also provide an instant meal with enhanced nutritional profile. Therefore, in this study, RSM was used to optimize the drum drying of brown rice‐based instant cereal blend containing anchovy powder using their physicochemical and rheological properties. This study would contribute to diversifying the utilization of anchovies, by using it to enrich an instant breakfast meal for improved nutrition.

## 2 MATERIALS AND METHODS

Fresh anchovies (*Engraulis* spp.) were obtained from a canoe at Tema fish landing site in Greater Accra, Ghana, and transported on ice to the food processing laboratory of CSIR‐Food Research Institute for processing. The batch of small fish, weighing 3.5 kg in total, was washed three times in freshwater before drying in an air oven (Apex B35E, England) at 60°C for 18 hr. After drying, the fish was manually beheaded and degutted, before milling into fine powder (400 μm). This was to ensure easy removal of the head and gut content and prevent spillage of gall, which may induce bitterness. The anchovy powder was packaged airtight in flexible HDPE bags, stored at 4°C, and used within one week. Brown rice was obtained from a local market and cleaned manually to remove foreign matter such as chaff and stones. The brown rice was subsequently milled into powder with a hammer mill (8″ Sprumaster, Christy and Norris Ltd., Surrey, UK), sealed airtight in flexible HDPE bags, and stored at room temperature for use within 1 week.

### 2.1 Sample preparation and drum drying

Brown rice and fish powders were mixed uniformly using a high‐speed blender (Waring E8420, Torrington, USA) to a final blend containing 5% fish powder. Based on the findings of a preliminary study, this proportion of fish powder does not have an unpleasant effect on taste or aroma of the final product. The blend was mixed into a slurry with water to a slurry solids concentration of 20%–40% (Table 1), ensuring homogeneity before drum drying. Drying of the slurry was done by manually dispensing onto a single drum dryer (ANDRITZ Gouda, The Netherlands). A schematic diagram of the single drum dryer is shown in Figure S1. One kilogram of slurry was dried for each factor combination. The dryer, consisting of a single steel drum (Ø 21”) with four roller applicators, was preheated to stabilize the temperature before introducing the slurry onto the drum surface. Drying temperature (110–130°C) was regulated by adjusting the input steam pressure. After traveling approximately ¾ of the drum's circumference, the dried product was scraped off the drum's surface by a doctor blade. The dried flakes were collected onto a stainless‐steel tray and left to cool to room temperature before milling in a laboratory blender (Waring E8420, Torrington, USA) into powder (425 μm) and packaging in transparent HDPE bags (250 g each) for further analyses.

**TABLE 1:** Process variables and their levels in the Box‐Behnken design

Independent variables	Code	Levels		
		Low	Middle	High
Drying Temperature (°C)	X_1_	110	120	130
Drum speed (rpm)	X_2_	5	10	15
Slurry solids concentration (%)	X_3_	20	30	40

### 2.2 Analyses of brown rice‐anchovy powder instant cereal

#### 2.2.1 Physicochemical and functional properties of brown rice‐anchovy powder instant cereal

##### Water activity

Water activity was determined on 10 g of powdered samples using a Rotronic HygroLab 2 water activity meter (Rotronic AG, Bassersdorf, Germany).

##### Color determination

Color of the powders was determined using a Minolta Chromameter (CR‐ 310 Minolta, Japan) previously calibrated with a reference white porcelain tile (L^0^ = 97.63, a^0^ = 0.31, and b^0^ = 4.63). The color was described by L* to depict the lightness/darkness of the powder using the D_65_ illuminant settings.

##### Bulk density

Bulk density was determined according to the method of Cai and Corke (2000) with slight modification. In brief, 10 g of the drum‐dried powder was weighed into a 100 ml graduated cylinder and gently tapped on a laboratory bench for 5 min. The final volume was recorded and used to calculate the bulk density in g/cm^3^.

##### Water absorption capacity and solubility index

Water Solubility Index (WSI) and Water Absorption Capacity (WAC) were determined using the method of Majzoobi et al. (2011). Briefly, 0.5 g of drum‐dried powder was mixed with 6 ml of distilled water for 30 min and centrifuged at 3,000 × g for 10 min. The supernatant was dried at 105°C for 4 hr. WSI and WAC were calculated as follows.

(1) $$\text{WSI}=\frac{\text{Weight of solids in dry supernatant}}{\text{Sample weight}}\times 100$$

(2) $$\text{WAC}=\frac{\text{Weight of wet sediment}}{\text{Sample weight}‐\text{weight of solids in dry supernatant}}\times 100$$

#### 2.2.2 Rheological and texture properties of brown rice‐anchovy powder instant cereal

##### Viscosity

Viscosity of reconstituted powders was measured using a Brookfield viscometer with an RV2 spindle (Model DV2T; Brookfield Engineering Labs, Inc., USA). Using an end condition of 4 min, viscosity data were recorded at 15 s intervals in multipoint averaging mode. Measurements were taken in a 250 ml beaker, 2 min after spindle was immersed and equilibrated in a 9% slurry of reconstituted drum‐dried powder. The torque was always maintained between 10% and 100% during replicates measurements at 10 rpm.

##### Texture analyses

Consistency and cohesiveness of reconstituted drum‐dried powder was evaluated using a back‐extrusion test with a texture analyzer (TA‐XT2 Plus Stable Micro Systems, Surrey, UK) equipped with 5 kg load cell and a perspex back‐extrusion rig. Seventy milliliters of porridge, obtained by reconstituting 12 g of drum‐dried powder in 100 ml of suspension and stirring gently to a smooth finish was filled into the perspex rig and compressed at a strain of 50% with a 40 mm plunger at a test speed of 1 mm/s. Numerical values of consistency and cohesiveness were measured by the Exponent Software (Stable Micro Systems, Surrey, UK).

##### Morphological features of brown rice‐anchovy powder instant cereal

Scanning Electron Microscopy (*SEM*) was used to visualize the morphological features of drum‐dried brown rice‐fish instant cereal. Samples, affixed to carbon taped stubs, were coated with gold in a vacuum evaporator (108 Manual Sputter Coater, Ted Pella Inc. USA). Electron micrographs of the gold‐coated samples were acquired using a Phenom Desktop *SEM* (Phenom‐World, The Netherlands) at an acceleration voltage of 15 kV.

### 2.3 Experimental design

To determine the effect of drum drying on the quality characteristics of the brown rice‐fish instant cereal, a Box‐Behnken design was adopted to optimize the processing factors. The independent variables drying temperature (X_1_ = 110, 120 and 130°C), drum speed (X_2_ = 5, 10 and 15 rpm), and slurry solids concentration (X_3_ = 20%, 30%, and 40%) were used to generate a 15‐point design matrix (STATGRAPHICS Centurion XVI, StatPoint Technologies Inc, USA) (Table 1).

### 2.4 Statistical analyses and optimization

A second‐order polynomial regression model was fitted to the results to assess the effect of the process variables on the various responses, at a significance level of *p* <.05. The following model was used.

(3) $$Y=\beta_{0}+\Sigma \beta_{i}x_{i}+\beta_{\mathrm{ii}}x^{2}+\Sigma \Sigma \beta_{\mathrm{ij}}x_{i}x_{j}$$

where *Y* represents a predicted response, β*_0_* is the interception coefficient, β*_i_* is the linear term, β*_ii_* are the quadratic terms, β*_ij_* are the interaction terms, and *x_i_* and *x_j_* represent coded levels of the independent variables, that is, drying temperature, drum speed, slurry solids concentration. Regression coefficient (*R^2^*) and a test for lack‐of‐fit were used to determine the adequacy of the regression models. Interaction between any two independent variables was visualized by surface plots in which the third variable was held constant (Statgraphics centurion XVI, StatPoint Tech. Inc, USA). Numerical optimization was performed using the desirability function, to find levels of the independent variables that gave the most suitable powder color, water activity, viscosity, and consistency of the reconstituted product. The optimization was verified by producing two separate batches of the product using the optimal conditions obtained from the experiment.

## 3 RESULTS AND DISCUSSIONS

Table 2 provides an overview of results obtained for the various assessments carried out on the drum‐dried products. These showed variations in individual responses because of different combinations of factors and factor levels for the various runs.

**TABLE 2:** Effect of processing variables on physical and functional properties of brown rice‐anchovy powder instant cereal

Run	Temperature (°C)	Drum Speed (rpm)	Solids Concentration (%)	Water activity	L*	Viscosity (cP)	Consistency (N.s)	Cohesiveness (*N*)	Bulk Density (g/cm^3^)	Water Binding Capacity (%)	Water Solubility Index (%)
1	120	10	30	0.503	63.49	276	1.3	−0.9	0.59	907.0	4.6
2	110	5	30	0.542	58.91	335	7.3	−0.6	0.56	923.6	4.1
3	130	5	30	0.215	57.41	242	8.0	−0.7	0.63	933.9	3.8
4	110	15	30	0.450	62.34	480	9.1	−0.9	0.53	958.1	3.8
5	130	15	30	0.216	59.94	242	3.1	−0.3	0.56	909.7	3.7
6	110	10	20	0.465	62.24	400	9.8	−0.9	0.53	852.8	1.6
7	130	10	20	0.181	61.81	470	6.4	−0.6	0.43	890.9	2.8
8	120	10	30	0.428	63.82	110	5.2	−0.2	0.53	824.9	2.8
9	110	10	40	0.497	64.02	792	2.3	−2.3	0.42	961.1	2.7
10	130	10	40	0.169	59.90	294	1.8	−1.9	0.67	802.7	3.1
11	120	5	20	0.543	61.47	1,224	3.7	−3.2	0.25	1,195.3	4.4
12	120	15	20	0.548	67.28	168	4.6	−0.2	0.72	876.2	2.8
13	120	5	40	0.538	57.70	248	2.0	−1.9	0.56	931.5	3.2
14	120	15	40	0.574	64.98	914	2.1	−2.2	0.37	1,021.5	4.2
15	120	10	30	0.494	61.20	572	1.2	−1.3	0.68	946.6	3.4

Regression coefficients of models describing the relationship between the processing variables and responses ranged between 85.7% and 98.1%, with none of the fitted models showing a significant lack‐of‐fit (*p* > .05) (Table 3). This indicates that the models adequately described the relationship between the independent and dependent variables.

**TABLE 3:** Estimated coefficients of fitted regression models for the responses

Variable	Water activity	L*	Viscosity (cP)	Bulk Density (g/cm^3^)	Consistency (N.s)	Cohesiveness (*N*)	Water Binding Capacity (%)	Water Solubility Index (%)
Constant	−21.76	−249.38	−9895.91	−5.57	458.01	38.39	−6219.6	−62.51
X_1_	0.3943^§§^	5.0844	216.11	0.0771	−7.527	−0.849	124.61	1.052
X_2_	−0.1031	1.7206^§^	−242.43	0.1550	3.150	0.255	−98.725	−1.038
X_3_	−0.0022	0.2449	−62.25	0.0475	−0.632^§^	0.653	19.61	0.459
X_1_^2^	−0.0017^§§^	−0.0203	−0.73	−0.0004	0.031^§^	0.003	−0.454	−0.004
X_1_X_2_	0.0004	−0.0045	−0.72	−0.0002	−0.033	0.003	−0.293	0.001
X_1_X_3_	−0.0001	−0.0092	−1.42	0.0001	0.007	0.001	−0.491	−0.002
X_2_^2^	0.0021^§^	−0.0464	3.15	−0.0016	0.048	−0.005	3.355^§^	0.026
X_2_X_3_	0.0002	0.0074	8.66^§^	−0.0033^§^	−0.004	−0.016	2.046^§^	0.013
X_3_^2^	0.0002	0.0118	2.43	−0.0013^§^	−0.006	−0.009	0.294	−0.006
Lack‐of‐ fit	0.721	0.382	0.775	0.292	0.651	0.447	0.564	0.787
*R* ^2^	98.10	85.73	87.18	89.67	83.00	81.61	84.83	71.60

Note

Values with a single section sign (§) are significant at .05, and values with a double section sign (§§) are significant at .01.

X_1_–Temperature (°C), X_2_–Drum speed (rpm), X_3_–Slurry solids concentration (%).

### 3.1 Physicochemical and functional properties of the drum‐dried cereal

**FIGURE 1:**
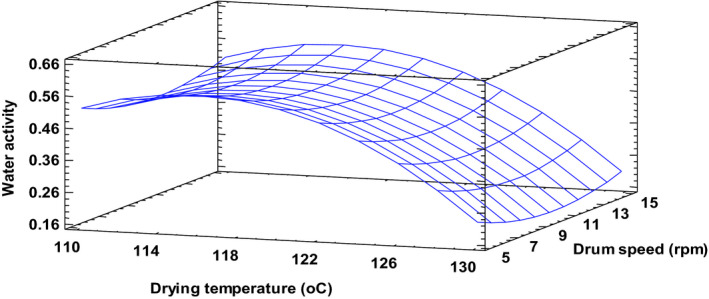
Water activity (a_w_) of drum‐dried cereal at a constant solids concentration of 40%. Fitted model: water activity (aw) = −21.7609 + 0.3944*X_1_ − 0.1031*X_2_ − 0.0022*X_3_ − 0.0017*X_1_^2^ + 0.0005*X_1_X_2_ − 0.0001*X_1_X_3_ + 0.0021*X_2_^2^ + 0.0002*X_2_X_3_ + 0.0002*X_3_^2^, where X_1_–Drying temperature, X_2_–Drum speed and X_3_–Slurry solids concentration

Water activity ranged between 0.2 and 0.6 for the various combination of process factors (Table 2). Low water activity is desirable for enhanced storage of dry food powders. The surface plot showing the relationship between processing parameters and water activity of the product (Figure 1) clearly exhibits the dependence of water activity on drying temperature. The water activity of the drum‐dried product decreased with increasing drying temperature. This was expected since higher drying temperatures enhance moisture removal (Fernando et al., 2008), however, beyond a certain moisture content, further increase in temperature may not have any marked effect on moisture content (Vallous et al., 2002). Regression analysis showed both linear and quadratic terms of temperature significantly (*p* < .05) affecting product water activity. Even though the linear term of drum speed did not have a significant effect on water activity, its quadratic effect was significant (*p* < .05), such that mid‐range drum speeds recorded much lower water activity compared to the extreme speeds. At high speeds cumulative heat flow over the drying cycle reduces, drum surface temperature decreases, and consequently moister flakes were obtained (Vallous et al., 2002). These trends were noticed for all levels of initial slurry solids concentration. Similar trends have been reported in previous studies by Kalogianni et al. (2002), Vallous et al. (2002), and Pua et al. (2010), in which higher speeds generally resulted in wetter products. There was no evidence to support the fact that more solids in the slurry result in moister final products as observed by Kalogianni et al. (2002). Differences in experimental conditions and slurry type may, at least, partly explain these discrepancies.

**FIGURE 2:**
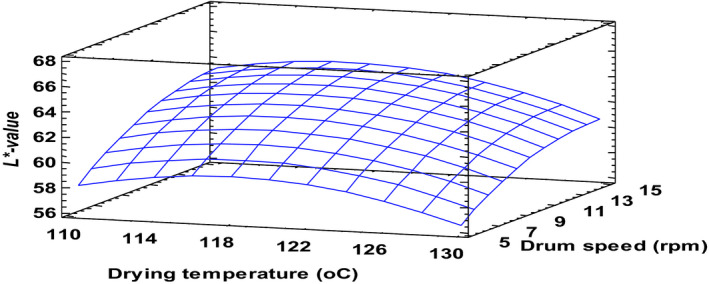
L *– value of drum‐dried cereal at a constant slurry solids concentration of 40%. Fitted model: L = −249.3770 + 5.0844*X_1_ + 1.7206*X_2_ + 0.2449*X_3_ − 0.0203*X_1_^2^ − 0.0045*X_1_X_2_ − 0.0092*X_1_X_3_ − 0.0464X_2_^2^ + 0.0074*X_2_X_3_ + 0.0118*X_3_^2^, where X_1_–Drying temperature, X_2_–Drum speed and X_3_–Slurry solids concentration

L*‐value, which represents the lightness or darkness of a product was influenced greatly by the drum speed compared to the other processing factors. The increase in drum speed resulted in an almost linear increment in L*‐value within the entire design space (Figure 2). This condition reduces thermal degradation of the product since drum surface temperature declines (Vallous et al., 2002). Lower drum speeds yielded products with darker color, and this is ascribed to longer residence times and the relatively higher drum surface temperature at slow drum speeds that Vallous et al. (2002) suggest. Pua et al. (2010) also reported an improvement in L*‐value of jackfruit powder when drum speed was progressively increased. Even though drying temperature did not significantly affect color, a general decrease in L*‐value occurred when temperature was gradually increased from 120 to 130°C. The trends in L*‐value may be explained mainly by nonenzymatic browning reactions such as Maillard and dextrinization, which may have been heightened as the product stayed longer on the heated drum or as temperature increased during processing (Abonyi et al., 2002; Nunes et al., 2020). This observation reflects those of Wiriyawattana et al. (2018), who also reported product darkening resulting from increased temperature during drum drying. The trends in L*‐value were identical for the different levels of solids concentration of the slurry.

**FIGURE 3:**
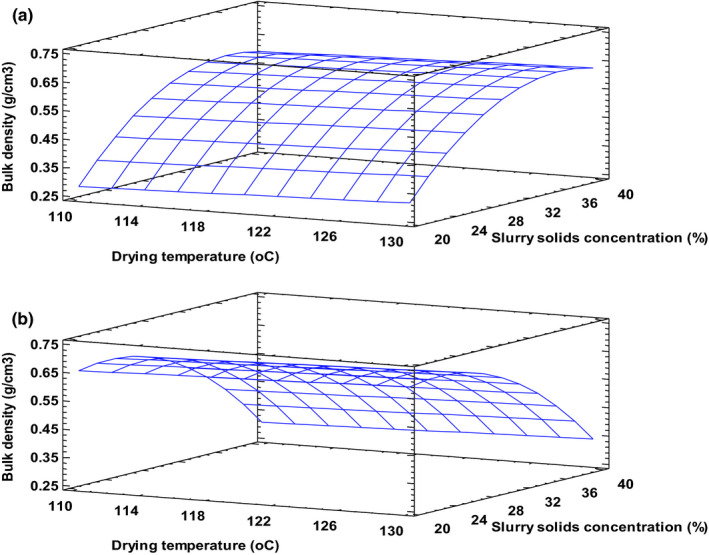
Bulk density at drum speed 5 rpm (a) and 15 rpm (b). Fitted model: Bulk density = −5.5718 + 0.0771*X_1_ + 0.1550*X_2_ + 0.0475*X_3_ − 0.0004*X_1_^2^ − 0.0002*X_1_X_2_ + 0.0005*X_1_X_3_ − 0.0016*X_2_^2^ − 0.0033*X_2_X_3_ − 0.0013*X_3_^2^, where X_1_–Drying temperature, X_2_–Drum speed and X_3_–Slurry solids concentration

**FIGURE 4:**
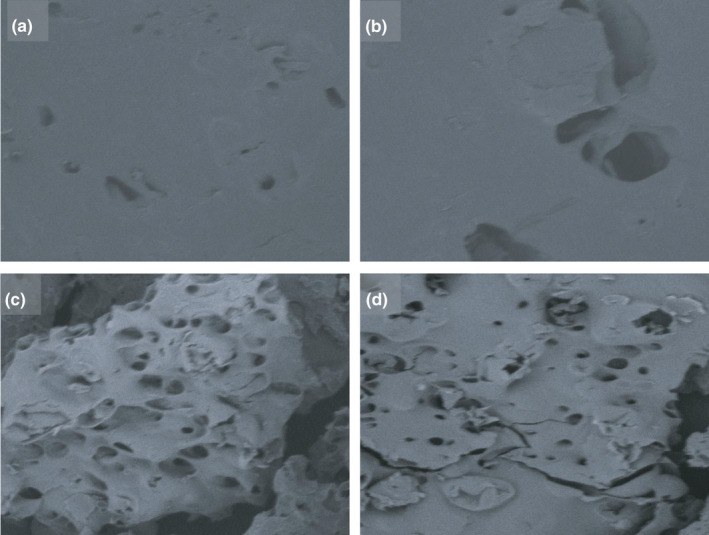
Scanning Electron Microscopy images of drum‐dried powder using Temperature (°C), Drum speed (rpm) and Slurry solids concentration (%) of (a) *Run 10*:130°C, 10 rpm, 40% (b) *Run 3*:130°C, 5 rpm, 30% (c) *Run 11*:120°C, 5 rpm, 20% (d) *Run 14*:120°C, 15 rpm, 40%

The bulk density of the drum‐dried powders ranged between 0.3 and 0.7 g/cm^3^, with a mean of 0.5 g/cm^3^. This is an important consideration in packaging, storage, transportation, and marketing of food powders. It is also a key determinant of powder flowability (Juliano et al., 2006). The values obtained were comparable to values obtained for instant rice porridge powder containing either soybean or mung bean (Mayachiew et al., 2015), complementary food from cereal, oilseed, and animal polypeptide (Fasuan et al., 2017) as well as a tuber‐based ready‐to‐eat food (Osei, 2019). The model for product bulk density explained nearly 90% of the variability in the experimental data. It showed that the main processing factors did not affect product bulk density. However, a significant interaction (X_2_X_3_) and quadratic effect (X_3_^2^) were observed to influence bulk density. Increasing levels of solids in the slurry had a reverse effect at the extreme drum speeds of 5 and 15 rpm (Figure 3A & B). Drying high solids slurry at low speeds resulted in thick dense flakes with high bulk density. Altan and Maskan (2012) explain that bulk density increases when incomplete gelatinization of starch occurs because high concentration of solids limits the amount of water available for gelatinization. It is also possible that this situation lowered the moisture evaporation rate leading to a product with low porosity (Figure 4A & B) and high bulk density. High drying temperature has been reported to reduce bulk density of some drum‐dried products because it enhances moisture evaporation rates and improves porosity (Jittanit et al., 2011). In this study, however, temperature had a negligible effect on bulk density.

**FIGURE 5:**
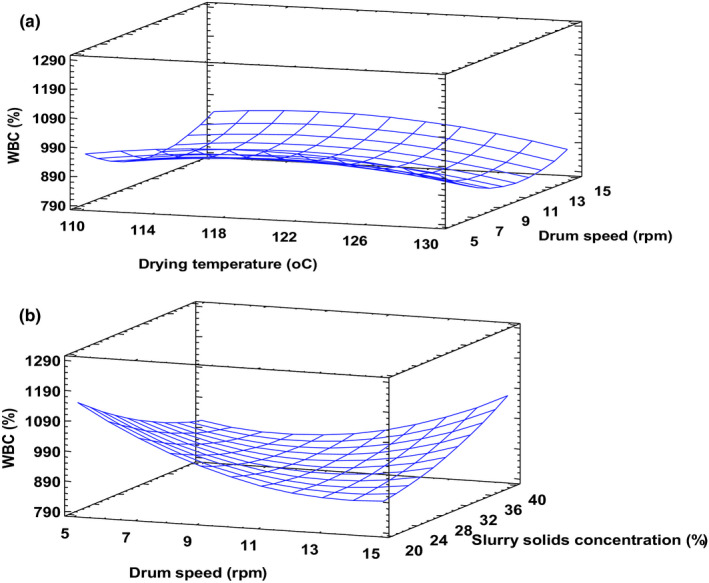
Water Binding Capacity (WBC) at slurry solids content 20% (a) and drying temperature 120°C (b). Fitted model: WBC = −6219.62 + 124.61*X_1_ − 98.7257*X_2_ + 19.6124*X_3_ − 0.453838*X_1_^2^ − 0.2934*X_1_X_2_^ ^− 0.4912*X_1_X_3_ + 3.3550*X_2_^2^ + 2.0456*X_2_X_3_ + 0.2941*X_3_^2^, where X_1_–Drying temperature, X_2_–Drum speed and X_3_–Slurry solids concentration

Hydration properties are important for processing and utilization of food powders. While WBC primarily depicts protein‐water interaction in food systems and is influenced by protein, starch, and fiber, WSI reflects the solubilization of starch when excess water is added (Oikonomou & Krokida, 2011). This affects digestibility and reflects the extent of molecular damage. The *R* ^2^ for this function explained more than 80% of the variability in the experimental data. Changes in product WBC were not greatly affected by temperature compared to drum speeds or slurry solids concentration (Figure 5A). A significant (*p* < .05) quadratic effect of drum speed and the interaction between drum speed and slurry solids concentration on WBC occurred (Figure 5B). The interaction between drum speed and slurry solids concentration revealed an interesting contrast in which a combination of low drum speed and low slurry solids concentration resulted in high WBC. These samples exhibited high porosity, as shown in Figure 4C & D. On the other hand, products obtained at the lowest level of slurry solids and highest speed had lower WBC, much the same way as the highest slurry solids content and lowest drum speed. This observation could be attributed to the fact that slow drum speed increases product exposure to heat, and this may cause extensive degradation of starch leading to low WBC (Soison et al., 2014). It is also possible that long resident time may have caused more dextrinization than gelatinization (Ding et al., 2006). Furthermore, prolonged exposure led to low porosity, especially at high slurry solids concentration (Figure 4A & B). Since WBC is related to the structure of food particles, changes that occur in particle structure or composition during processing may lead to changes in WBC (Laufenberg & Schulze, 2009). WBC of this product were higher than the range of 39%–96% reported for a cereal‐based complementary food (Fasuan et al., 2017).

**FIGURE 6:**
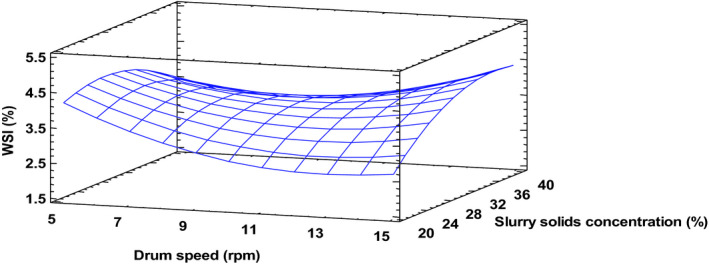
Water solubility index at drying temperature 120°C. Fitted model: WSI = −62.5098 + 1.0518*X_1_^ ^− 1.0381*X_2_ + 0.4593*X_3_^ ^− 0.0041*X_1_^2^ + 0.0010*X_1_X_2_^ ^− 0.0016*X_1_X_3_ + 0.0259*X_2_^2^ + 0.0126*X_2_X_3_ − 0.0062X_3_^2^, where X_1_–Drying temperature, X_2_–Drum speed and X_3_–Slurry solids concentration

WSI in drum‐dried products is ascribed to the destruction of starch granules and reduction in crystallinity. Results of the present study showed that both slurry solids content and residence time affected WSI of the drum‐dried product (Figure 6). Whereas increasing slurry solids concentration from 20% to 40% intensified the WSI, increasing drum drying speed had a contrary effect. When slurry solids concentration increases, WSI increases because many more starch granules are available for degradation by heat and shear between starch granules and drum surface (Supprung & Noomhorm, 2003). An increase in drum speed restricts the exposure time of the product to heat, therefore, limiting the extent of starch molecular damage and WSI accordingly. WSI recorded in this study was generally lower compared to results obtained for low amylose rice by Supprung and Noomhorm (2003), rice berry flour by Wiriyawattana et al. (2018), purple sweet potato flour (Soison et al., 2014), and complimentary food (Eshun, 2019). It is possible that some extent of crosslinking may have occurred between starch and protein (mainly from anchovy powder), leading to restrict starch degradation and reduced WSI (Matthey & Hanna, 1997).

### 3.2 Rheological properties of the drum‐dried cereal

Rheological properties of instant foods are important in terms of consumer response and product characterization. These greatly affect the eating quality of food and constitute an important basis for classification of food. In this study, the rheological properties assessed were viscosity, consistency, and cohesiveness. The product viscosity ranged between 110 and 1,224 cP.

**FIGURE 7:**
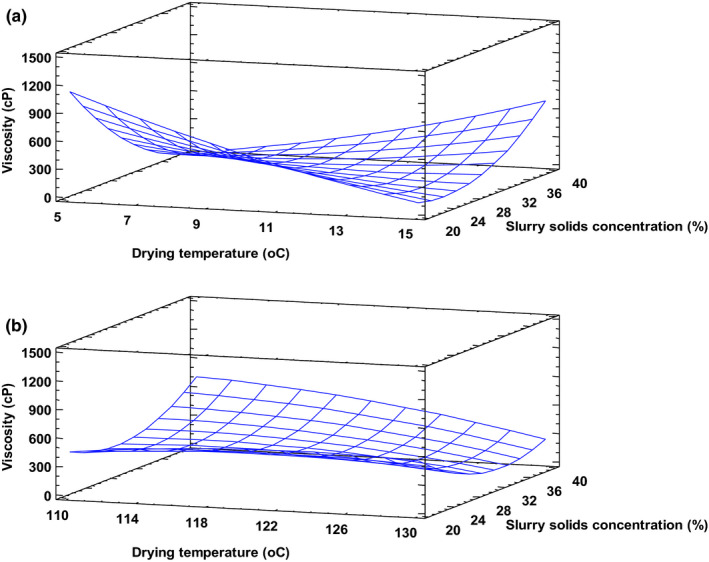
Viscosity of drum‐dried cereal at a constant drying temperature of 130°C (a) and at constant drum speed of 10 rpm (b). Fitted model: Viscosity = −9895.91 + 216.1170*X_1_ − 242.4320*X_2_ − 62.2450*X_3_ − 0.7324*X_1_^2 ^− 0.7230*X_1_X_2_^ ^− 1.4200*X_1_X_3_ + 3.1503*X_2_^2^ + 8.6600*X_2_X_3_ + 2.4291*X_3_^2^, where X_1_–Drying temperature, X_2_–Drum speed and X_3_–Slurry solids concentration

None of the linear and quadratic terms had a significant effect on product viscosity. The interaction between drum speed and slurry solids concentration, however, had a significant impact on viscosity. For instance, at 5 rpm, increased slurry solids concentration caused a drastic reduction in product viscosity (Figure 7A), but this seemed to taper off around intermediary drum speed. Beyond this point, a reversed trend is noticed, in which increase in both drum speed and slurry solids concentration resulted in a more viscous end‐product. This observation is in accordance with Supprung and Noomhorm (2003), who explained that at high solids content there is shearing and pressing of granules against the drum surface, leading to increased granule degradation. However, increase in drum speed may have reduced the rate of granule disruption and shearing, and reversed the trend in viscosity. Anastasiades et al. (2002) explain that the chemical structure of starch does not change during drum drying, rather, starch components degrade into lower molecular weight macromolecules, leading to changes in viscosity. Generally, temperature did not have much effect on the viscosity of drum‐dried product (Figure 7B).

**FIGURE 8:**
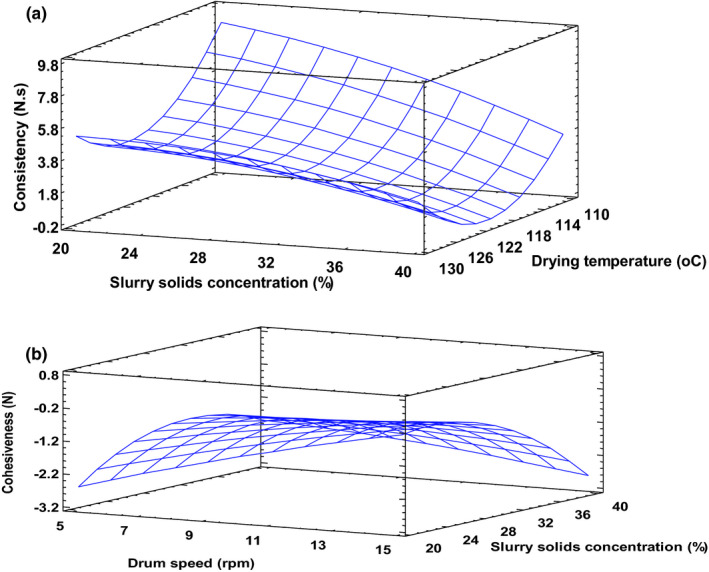
(a) Consistency at drum speed 10 rpm. Fitted model: Consistency = 485.005 − 7.5266*X_1_ + 3.1502*X_2_ − 0.6315*X_3_ + 0.0314*X_1_^2^ − 0.0334*X_1_X_2_ + 0.0071*X_1_X_3_ + 0.0468*X_2_^2^ − 0.0041*X_2_X_3_^ ^− 0.0065X_3_^2^, where X_1_–Drying temperature, X_2_–Drum speed and X_3_–Slurry solids concentration. (b): Cohesiveness at drying temperature 120°C. Fitted model: Cohesiveness = 38.3890 − 0.8488*X_1_ + 0.2554*X_2_ + 0.6529*X_3_ + 0.0035*X_1_^2^ + 0.0033*X_1_X_2_ − 0.001*X_1_X_3_ − 0.0051*X_2_^2^ + 0.0162*X_2_X_3_ − 0.0090X_3_^2^, where X_1_–Drying temperature, X_2_–Drum speed and X_3_–Slurry solids concentration

Consistency is associated with viscosity whereas cohesiveness describes the tendency of a product to stick together. The models for consistency and cohesiveness explained about 93% and 82% of the variability in the experimental data for these two attributes correspondingly. Slurry solids concentration and drum drying temperature markedly affected consistency of the reconstituted product. Increasing slurry solids concentration almost linearly reduced consistency, while extreme levels of temperature (especially the lowest temperature) resulted in increased product consistency (Figure 8A). This trend occurred for all levels of drum rotation speed rates. In addition to high granule degradation explained earlier, another possible reason could be the low porosity of samples whose slurry contained high concentration of solids and dried at high temperature (as shown in Figure 4A & B), while high porosity was obtained using the midpoint drying temperature (Figure 4C & D). Additionally, at higher slurry solids concentration more granules are available for degradation at high drying temperatures (Liu et al., 2013; Supprung & Noomhorm, 2003). Therefore, when reconstituted, they are unable to hydrate extensively into a slurry of thick consistency. Cohesiveness, on the other hand, reduced linearly with increasing drum speed and beyond the mid‐range slurry solids concentration (Figure 8B).

### 3.3 Optimization

Numerical multiple‐response optimization was performed to determine the ideal combination of process parameters to produce the most desirable brown rice‐based instant cereal containing anchovy powder. The criteria for optimization were water activity (min), bulk density (min), L*‐value (max), viscosity (min), consistency (max), and WBC (max). The optimized conditions were temperature of 130.0°C, drum speed of 9.3 rpm, and a slurry solids concentration of 20.5%, with a desirability of 0.718. To validate the adequacy of the predicted models, the optimum processing conditions were used to produce the instant cereal enriched with anchovy powder and predicted values of the indices compared to the experimental data. The results showed that the experimental values deviated marginally from the predicted values (Table 4), authenticating the predicted models obtained in the study.

**TABLE 4:** Predicted and observed values of brown rice‐anchovy powder instant cereal produced using the optimum processing conditions

Variable	Predicted	Observed	Deviation^§§^
Water activity	0.184	0.200	0.02
L*	62.17	62.33	0.003
Viscosity (cP)	573	581	0.01
Bulk density (g/cm^3^)	0.44	0.40	0.10
Consistency (N.s)	5.50	5.21	0.06
WBC (%)	932.5	948	0.02

^§§^ Deviation = (observed−predicted)/observed.

## 4 CONCLUSIONS

The study shows a complex relationship between drying temperature, drum speed, and slurry solids concentration, with respect to their individual as well as combined effect on product characteristics of brown rice‐anchovy powder instant cereal. Each of these factors or their combination affected the product quality differently. High drying temperature resulted in reduced water activity, darker product, and reduced consistency. Drum speed greatly affected (*p* < .05) product luminosity and WBC, while slurry solids concentration affected bulk density and consistency of the reconstituted product. The response surface models were adequate to describe these relationships, with *R* ^2^ > 70%, and efficient in process optimization, leading to an optimal temperature of 130°C, drum speed 9.3 rpm, and slurry solids concentration of 20.5% for drying brown rice‐based instant cereal containing anchovy powder, using a single drum dryer. The study shows that anchovies are suitable for inclusion in cereal‐based ready‐to‐eat products and might improve the micronutrients content of the product.

## CONFLICT OF INTEREST

The authors declare that there is no conflict of interest.

## AUTHOR CONTRIBUTIONS

**Paa T. Akonor:** Conceptualization (equal); Formal analysis (equal); Investigation (equal); Methodology (equal); Writing‐original draft (lead); Writing‐review & editing (equal). **Amy Atter:** Conceptualization (equal); Investigation (equal); Funding acquisition (equal); Methodology (equal); Project administration (equal); Writing‐original draft (equal); Writing‐review & editing (equal). **Margaret Owusu:** Conceptualization (equal); Investigation (equal); Methodology (equal); Writing‐original draft (equal); Writing‐review & editing (equal). **Jonathan Ampah:** Conceptualization (equal); Investigation (equal); Methodology (equal); Writing‐original draft (equal); Writing‐review & editing (equal). **Anthonia Andoh‐Odoom:** Conceptualization (equal); Investigation (equal); Methodology (equal); Writing‐original draft (equal); Writing‐review & editing (equal). **Ragnhild Overå:** Funding acquisition (equal); Project administration (equal); Writing‐review & editing (equal). **Marian Kjellevold:** Funding acquisition (equal); Project administration (equal); Writing‐review & editing (equal). **Johannes Pucher:** Funding acquisition (equal); Project administration (equal); Resources (equal); Writing‐review & editing (equal). **Jeppe Kolding:** Funding acquisition (equal); Project administration (equal); Writing‐review & editing (equal).

## Supporting information

Figure S1

## Data Availability

Data sharing is not applicable to this article as all data are presented in the study.
