# Supplementary material for: Anchovy powder enrichment in brown rice‐based instant cereal: a process optimization study using Response Surface Methodology (RSM)
Source: Food Sci Nutr. 2021 Jun 30;9(8):4484–96. doi: 10.1002/fsn3.2424 (PMC8358353; doi:10.1002/fsn3.2424)
Supplement: Supplementary file 1 — Figure S1 [file FSN3-9-4484-s001.docx]

Applicator roller

Drum

Blade

Steam

Collector

Supplementary Figure 1: Schematic representation of the single drum dryer used in the study
